# Synthesis of Abscisic Acid in *Neopyropia yezoensis* and Its Regulation of Antioxidase Genes Expressions Under Hypersaline Stress

**DOI:** 10.3389/fmicb.2021.775710

**Published:** 2022-01-10

**Authors:** Jiali Yang, Wenhui Gu, Zezhong Feng, Bin Yu, Jianfeng Niu, Guangce Wang

**Affiliations:** ^1^Key Laboratory of Experimental Marine Biology, Institute of Oceanology, Chinese Academy of Sciences (IOCAS), Qingdao, China; ^2^Laboratory for Marine Biology and Biotechnology, Pilot National Laboratory for Marine Science and Technology, Qingdao, China; ^3^Center for Ocean Mega-Science, Chinese Academy of Sciences (CAS), Qingdao, China; ^4^College of Earth and Planetary Sciences, University of Chinese Academy of Sciences, Beijing, China; ^5^Marine Science and Engineering College, Qingdao Agricultural University, Qingdao, China

**Keywords:** abscisic acid, antioxidase, ABRE motif, ABA signaling pathway, *Neopyropia yezoensis*

## Abstract

Abscisic acid (ABA) is regarded as crucial for plant adaptation to water-limited conditions and it functions evolutionarily conserved. Thus, insights into the synthesis of ABA and its regulation on downstream stress-responsive genes in *Neopyropia yezoensis*, a typical Archaeplastida distributed in intertidal zone, will improve the knowledge about how ABA signaling evolved in plants. Here, the variations in ABA contents, antioxidant enzyme activities and expression of the target genes were determined under the presence of exogenous ABA and two specific inhibitors of the ABA precursor synthesis. ABA content was down-regulated under the treatments of each or the combination of the two inhibitors. Antioxidant enzyme activities like SOD, CAT and APX were decreased slightly with inhibitors, but up-regulated when the addition of exogenous ABA. The quantitative assays using real-time PCR (qRT-PCR) results were consistent with the enzyme activities. All the results suggested that ABA can also alleviate oxidative stress in *N. yezoensis* as it in terrestrial plant. Combined with the transcriptome assay, it was hypothesized that ABA is synthesized in *N. yezoensis* via a pathway that is similar to the carotenoid pathway in higher plants, and both the MVA and that the MEP pathways for isoprenyl pyrophosphate (IPP) synthesis likely exist simultaneously. The ABA signaling pathway in *N. yezoensis* was also analyzed from an evolutionary standpoint and it was illustrated that the emergence of the ABA signaling pathway in this alga is an ancestral one. In addition, the presence of the ABRE motif in the promoter region of antioxidase genes suggested that the antioxidase system is regulated by the ABA signaling pathway.

## Introduction

Abscisic acid (ABA) is a phytohormone that is found in all photosynthetic organisms (Cutler and Krochko, 1999; Hunter, 2007). In higher plants, ABA is known as a stress related hormone and plays a critical role in the regulation of various stress responses, including cold, salt, and drought stresses (Nogueira et al., 2003; Tijero et al., 2016; Jiang et al., 2019). The environmental stress factors mentioned above inevitably cause oxidative damage to cells (Kaur and Pati, 2016). Guan et al. (2000) discovered that exogenously applied ABA and H_2_O_2_ reinforced the expression of the antioxidant *Cat1* gene in maize and proposed that H_2_O_2_ was an intermediary that was involved ABA regulation of *Cat1* gene expression during osmotic stress. Jiang and Zhang (2002) also reported that ABA levels significantly increased under water stress in maize leaves. Furthermore, reactive oxygen species (ROS), such as O_2_^–^ and H_2_O_2_, and antioxidant enzymes, such as superoxide dismutase (SOD), catalase (CAT), ascorbate peroxidase (APX), and glutathione reductase (GR), were also strongly up-regulated after 12 h of osmotic stress. Recently, several studies have reported that ROS levels increased under alkaline osmotic stress in higher plants, and that the application of ABA effectively reduced ROS levels. Other studies have shown that ABA significantly enhanced SOD, CAT, peroxidase (POD), and APX activity (Sahu and Kar, 2018; Liu et al., 2019).

It is believed that ABA contents in algae are usually lower than in higher plants, but it plays a more important role in physiological activity (Huang et al., 2010; Yokoya et al., 2010). Hirsch et al. (1989) determined the ABA contents in 64 algal species and found that its content increased under hyperosmotic salinity stress. The oxidative stress response has been a very active research field, not only in higher plants, but also in algae. Under oxidative stress, ABA is involved in regulating algal morphogenesis during the transformation from vegetative to cyst cells in the unicellular green alga *Haematococcus pluvialis* (Kobayashi et al., 1997). Yoshida et al. (2003) added exogenous ABA to *Chlamydomonas reinhardtii* and measured the changes to key enzyme activities in the antioxidant system and found that SOD, CAT, and APX activities significantly increased.

The stress responsive pathway mediated by ABA has been deciphered in the model plant *Arabidopsis thaliana*. It is usually controlled by several core components, namely, ABA receptors (PYLs), type 2C protein phosphatases (PP2Cs), and class III sucrose-non-fermenting 1 (SNF1)-related protein kinase 2 (SnRK2). In the presence of ABA, PYL protein binds to PP2C and prevents activity. Thus, SnRK2 is released and the phosphorylated SnRK2 activates the ABA response element-binding factors (ABFs), which initiates downstream expression of ABA-responsive element genes in an ABRE-dependent manner (Soon et al., 2012; Fujita et al., 2013). Collectively, the ABA signaling pathway belongs to a sophisticated and intricate responsive pathway and it seems to be conserved across plants, including higher plants, bryophytes, and algae (Komatsu et al., 2020). However, parts of these core components have not been reported in algae (Komatsu et al., 2020).

The same is true for the synthesis of ABA (Hauser et al., 2011). The first biosynthetic pathway for ABA was discovered in a plant mutant (Linforth et al., 1987; Taylor et al., 1988). Since then, ABA biosynthesis and its metabolic pathways in higher plants have been extensively studied. Now, it is generally accepted that there are two ABA synthesis pathways, including the carotenoid pathway (indirect pathway) which is present in higher plants, and the direct pathway, which is mainly found in two fungi, *Cercospora rosicola* and *Cercospora cruenta* (Bennett et al., 1984; Inomata et al., 2004; Takino et al., 2019). In both pathways, ABA is derived from the five carbon precursor, isoprenyl pyrophosphate (IPP) (Nambara and Marion-Poll, 2005). Isoprenyl pyrophosphate can be biosynthesized *via* two pathways: (1) the mevalonate (MVA) pathway, where it is synthesized in the cytoplasm using acetyl-CoA as a substrate, and (2) the methylerythritol phosphate (MEP) pathway, where it is synthesized in the plastids using pyruvate and glyceraldehyde 3-phosphate (GA-3P) as substrates (Jomaa et al., 1999; Lichtenthaler, 2000; Yang et al., 2012). The MEP pathway is the main synthesis pathway for the IPP in higher plants and also operates in bacteria and green algae (Hunter, 2007; Phillips et al., 2008). Several studies have shown that 1-deoxy-D-xylulose 5-phosphate synthase (DXS) is one of the most important rate-limiting enzymes in the MEP pathway (Estevez et al., 2001; Cordoba et al., 2011), and it can be inhibited by clomazone (2-[(2-chlorophenyl) methyl]-4,4-dimethyl-3- isoxazolidinone) (Carretero-Paulet et al., 2006; Han et al., 2013). However, the MVA pathway, mainly found in the cytoplasm, is the only synthetic pathway for IPP in many fungi and animals. In this pathway, 3-hydroxy-3-methylglutaryl coenzyme A reductase is the rate-limiting enzyme and it can be inhibited by mevinolin (Mev) (Jomaa et al., 1999). Studies have suggested that some green algae, such as *C. reinhardtii* and *Scenedesmus obliquus*, only have an MEP pathway (Schwender et al., 1996; Disch et al., 1998). However, there are different opinions about red algae (Rhodophyta) (Schwender et al., 1997; Grauvogel and Petersen, 2007), especially *N. yezoensis* where the IPP synthesis pathway has not yet been elucidated.

*Neopyropia yezoensis* is a typical intertidal macroalgae that belongs to the Bangiales and represents an ancient lineage of red algae with simple morphology (Sutherland et al., 2011). At low tide, *N. yezoensis* lose as much as 90% of their cellular water due to desiccation stress (Blouin et al., 2011), but their activities can completely recover when rehydrated. Therefore, *N. yezoensis* has been suggested as a model organism for investigating the stress tolerance mechanisms in intertidal seaweed (Blouin et al., 2011; Sun et al., 2015). Previous results showed that the activities of some antioxidant enzymes were significantly up-regulated under hypersaline stress (Yu et al., 2020). However, it is not known whether ABA is involved in regulating the expression of antioxidant enzymes.

Here, we applied exogenous ABA to *N. yezoensis* thalli and/or treated them with two specific inhibitors of ABA synthesis. Then the photosynthetic parameters, the ABA content, and the variation in antioxidant enzymes activity were determined under high salinity stress conditions (120‰). The samples under the designed conditions were collected and subjected to a transcriptome analysis. Based on the results, a theoretical synthesis pathway for ABA was analyzed and the possible regulation of related antioxidant enzymes by ABA was investigated. These results improve our understanding of ABA-mediated stress resistance in *N. yezoensis* and will improve understanding about the evolution of the ABA signaling pathway. The results also provide a theoretical foundation for the cultivation of new strains with excellent resistance.

## Materials and Methods

### Algae Preparation

The *N. yezoensis* thalli samples were collected from an algae culture farm in the offshore cultivation area of Jimo, Shandong Province, China. The selected algae were cultured in fresh seawater with PES medium at 10°C under 50 μmol photons m^–2^ s^–1^ light provided by halogen lamps with a photoperiod of 12 h light/12 h dark. The culture medium was constantly filled with filter-sterilized air and replaced every day.

### Inhibitor and Hyperosmolarity Stress Treatments

The stock solutions of inhibitors clomazone (Clo) and mevinolin (Mev) were prepared in DMSO at concentration 50 and 25 μM, respectively, in the dark at 4°C. The ABA was dissolved in methanol to a concentration of 100 mg mL^–1^ and stored at −20°C with light preservation. All the stock solution was diluted 1,000 times before it was used in the inhibitor treatment experiments. The 120‰ high salinity seawater was prepared by adding the sea salt to fresh seawater and the salinity was monitored by a salinity meter.

Fresh seaweed samples were treated in 120‰ salinity seawater at 10°C under 50 μmol photons m^–2^ s^–1^ of artificial light for 1 h. Then, the high salinity treated samples were recovered in seawater containing the one or both of the inhibitors, or the two inhibitors combined with ABA for 4 h. This procedure allowed the inhibitors to enter the algae when it was rehydrated after the high salinity treatment. The control (C) was just subjected to the high salinity treatment and was recovered in normal seawater. About 1 g (fresh weight) recovered *N. yezoensis* of each treatment was treated again with 120‰ salinity seawater containing the chemicals in subsequent experiments (Supplementary Figure 2). All the samples under each treatment were collected separately, immediately frozen with liquid nitrogen, and stored at −80°C. Three parallel samples were processed for each treatment.

### Determination of the Photosynthetic Parameters

The recovered *N. yezoensis* was treated again with 120‰ salinity seawater containing the different chemicals for 4 h and rehydrated for another 4 h. The photosynthetic parameters for the samples after 4 h dehydration (S4h), and 0.5, 2, or 4 h of rehydration (R0.5h, R2h, and R4h, respectively) were determined using a Dual-PAM-100 measuring system (Heinz Walz, Effeltrich, Germany). All the samples were kept in the dark for 10 min before determining the chlorophyll fluorescence parameters. The procedure followed (Xie et al., 2020) and used actinic light at 57 μmol m^–2^ s^–1^. Maximal PS II quantum yield, *F*_*v*_/*F*_*m*_, and the quantum yield of non-regulated energy dissipation in PS II [Y(NO)] were automatically calculated by the software provided. Here, Y(NO) values were increased indicated that the algae had been damaged to a certain extent, and a decrease indicates that the algae has recovered.

### Determination of the ABA Content in the Samples

Four inhibitor treatments, which were Clo, Mev, Clo + Mev (MC), and Clo + Mev + ABA (CMA) were applied to explore the effects of the inhibitors on ABA content. The treated samples (about 100 mg fresh weight) were frozen in liquid nitrogen and ground by grinders (Jingxin Technology, Shanghai) in 2 ml grinding tubes. A total of 10 ng d6-ABA was added to monitor the ABA losses from the samples during extraction and purification (Dobrev et al., 2005). Next, 500 μL ABA extraction solvent (methanol:H_2_O:CH3COOH = 89:10:1, vol/vol/vol) was added and incubated for 30 min at 120 rpm at 4°C. The supernatant was obtained through centrifugation at 13,000 *g* for 5 min at 4°C and an equal volume of petroleum ether was added to remove the components affecting ABA determination. Then, the ABA crude extract was enriched and eluted through a C_18_-SPE column (Waters, Milford, MA, United States) (Oh et al., 2018) and the elute was concentrated with pre-cooled nitrogen (not completely dried to avoid any ABA losses). The ABA samples were made up to 50 μL using methanol:H_2_O:CH3COOH = 20:79:1 (vol/vol/vol). Finally, the obtained ABA extract was injected into an HPLC–MS/MS system (Bruker, Compact II, Germany) and determined (Lopez-Carbonell et al., 2009; Pan et al., 2010; Fu et al., 2012).

### Determination of the H_2_O_2_ Concentration in Each Sample

Based on the ABA content results for each sample, the MC, CMA and the control (C) were further selected to investigate the effects of ABA on the expression of antioxidant enzymes. Firstly, the H_2_O_2_ contents in the C, MC and CMA treated *N. yezoensis* thalli were determined using the corresponding assay kits from Sigma-Aldrich (St. Louis, MO, United States). The samples were extracted according to Yu et al. (2020).

### Activities of Enzymes Associated With the Anti-oxidative System

The SOD, CAT, and GPX enzyme activities in the C, MC, and CMA treated *N. yezoensis* thalli were determined using the corresponding assay kits from Sigma-Aldrich. The APX, POD, and NOX activities were determined using an assay kit supplied by Jian Cheng (Nanjing Jiancheng Institute of Biological Engineering, China). The protein extractions were carried out according to Yu et al. (2020).

### RNA Extraction, cDNA Library Construction, Sequencing and Annotation

The MC, CMA, and C samples were used to carry out the transcriptome assay. The total RNA from each sample was extracted using an RNAprep Pure Plant kit (Tiangen, Beijing, China) according to manufacturer’s instructions. The RNA integrity was checked by 1% agarose gel electrophoresis, and the concentration and purity of the RNA was detected by a NanoPhotometer NP80 Touch (Implen Gmbh, Munich, Germany). A total of 3 μg RNA was used to construct the cDNA library, which was sequenced on an Illumina HiSeq 2500 system at Wuhan Frasergen Bioinformatics Technology Co., Ltd., Wuhan, China.

The expression level of the genes was ascertained from the fragments per kilobase per million bases (FPKM) value, and screened using RSEM and bowtie2 (Trapnell et al., 2010; Li and Dewey, 2011). DESeq2 (1.22.2) was used to determine the differentially expressed genes (DEGs) and all DEGs reached the conditions of FDR < 0.05, log2FC > 1, or log2FC < −1 (Love et al., 2014).

Functional annotation of the DEGs was performed by Blast^1^ against the NCBI non-redundant protein sequences (NR), Kyoto Encyclopedia of Genes and Genomes (KEGG), Swiss-Prot, Gene Ontology (GO), and Clusters of euKaryotic Orthologous Groups of proteins (KOG) databases. The statistical enrichment analyses of DEGs in the KEGG pathway were performed using KOBAS software (Mao et al., 2005) and FDR values less than or equal to 0.05 were defined as differentially expressed metabolic pathways.

### Validation of Gene Expression Using the Quantitative Assays Using Real-Time PCR Method

Based on the variation recorded for the annotated transcripts, nine DEGs, consisting of three genes related to the biosynthesis of ABA and six enzyme genes associated with the anti-oxidative system, were selected to perform the quantitative assays using real-time PCR (qRT-PCR). The feasibility of the transcriptome data was validated, and the effects of inhibitors on the synthesis of ABA precursors and the expression of antioxidant enzymes under hypersaline conditions were studied. The *Actin* gene was used as the internal control (Supplementary Table 1). The qRT-PCR assays were carried out using 2× SG Fast qPCR Master Mix (High Rox, B639273, Japan) on a StepOne Plus Multicolor Real-Time PCR Detection System (ABI, Foster City, CA, United States). Each gene in the different samples was determined using three technical replicates. The relative gene expression value was calculated by the 2^–ΔΔCt^ method (Livak and Schmittgen, 2001).

### Analysis of the Core Components of the Abscisic Acid Signaling Pathway in *N. yezoensis*

Abscisic acid is regarded as crucial for plant adaptation to water-limited conditions and it functions evolutionarily conserved among terrestrial plants, bryophytes, and algae. The Rhodophyta, *N. yezoensis*, is a typical Archaeplastida that is found in intertidal zone, which represents a transition habitat for plant evolution and colonization of terrestrial environments. Thus, we screened the sequence information about core components of the ABA signaling pathway from the transcriptome data and made comparisons with those published in other species. Since ABA activates the expression of downstream target genes by binding AREB to the ABA-responsive elements (ABREs) in the promoter regions, we also investigated the target sequence for AREB on the promoter of several antioxidase genes, including *SOD, CAT, APX, GPX*, and *POD*.

### Data and Statistical Analysis

All experimental data were derived from three independent measurements (±SD). Statistical analysis was performed using SPSS 26.0^2^, and significances differences were determined using analysis of variance (Duncan’s test; *P* < 0.05).

## Results

### Changes to the Photosynthetic Parameters Under the Different Treatments

**FIGURE 1:**
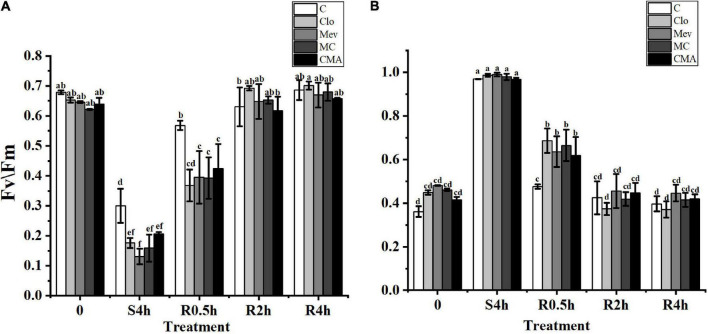
The change of *F*_*v*_/*F*_*m*_ **(A)** and Y(NO) **(B)** in different treatments of *Neopyropia yezoensis*. The abscissa, respectively, represented before hypersaline stress (0), under severe high salinity (120‰) stress 4 h (S4h), normal seawater recovery at 0.5, 2, and 4 h with different inhibitor treatments (R0.5h, R2h, and R4h). C, control; Clo, clomazone; Mev, mevinolin; MC, Mev + Clo; CMA, Clo + Mev + ABA. *F*_*v*_/*F*_*m*_, maximal PS II quantum yield; Y(NO), quantum yield of non-regulated energy dissipation. Data were Mean ± SD (*n* = 3). Different letters indicate significant differences (*P* < 0.05).

The *F*_*v*_/*F*_*m*_ and Y(NO) values for the control, the Clo, Mev, MC, and CMA chemical treatments showed different trends. Before hypersaline stress treatment, *F*_*v*_/*F*_*m*_ was not significantly different among the samples (*P* > 0.05) (Figure 1A). However, after severe high salinity (120‰) stress for 4 h (S4h), the *F*_*v*_/*F*_*m*_ value for each sample decreased to about 42.1, 27.0, 20.3, 25.6, and 36.4%, respectively, of the values recorded for the algae before hypersaline stress. During rehydration, all the *F*_*v*_/*F*_*m*_ values in the different treatments recovered rapidly, but the recovery speed for the samples treated with inhibitors was slower than the control. At R2h, the *F*_*v*_/*F*_*m*_ values of all the samples were restored to their original levels before the treatments.

The Y(NO) values for the different inhibitor treatments were higher than that of the control before hypersaline stress (Figure 1B). At S4h, the Y(NO) values in all treatments were close to 1. However, there were no statistically significant differences between the ABA addition treatment (CMA) and the other treatments (Figure 1B). In a similar way to *F*_*v*_/*F*_*m*_, Y(NO) also rapidly decreased when the samples were subjected to normal seawater, but the recovery period was longer in the inhibitor treatments.

### Impact of Inhibitors on Abscisic Acid Content Under High-Salinity Stress

**FIGURE 2:**
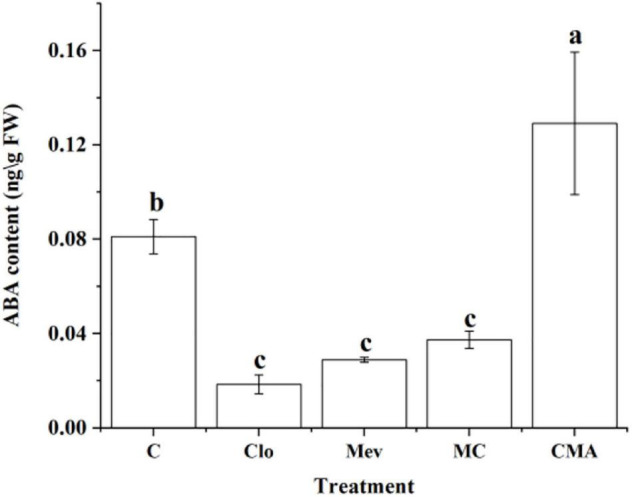
Abscisic acid (ABA) content under hypersaline (120‰) stress 4 h (S4h) in *N. yezoensis* after different inhibitor treatments. C, treatment of 120‰ hypersaline stress; Clo, clomazone treatment under hypersaline stress; Mev, mevinolin treatment under hypersaline stress; MC, Mev and Clo treatment under hypersaline stress; CMA, Clo, Mev and ABA treatment under hypersaline stress simultaneously. Data were Mean ± SD (*n* = 3). Different letters indicate significant differences (*P* < 0.05).

The ABA content was 0.082 ng g^–1^ under 120‰ hypersaline stress only (control, C) (Figure 2). However, it was 0.019, 0.029, and 0.037 ng g^–1^ after the Clo, Mev, and MC treatments, respectively. Therefore, ABA level decreased significantly (*P* < 0.5) under the different inhibitor treatments by 2–4 times compared to C (Figure 2). While adding of exogenous ABA (CMA treatment) significantly increased the ABA content to almost 1.5 times of that in the control.

### Variations in H_2_O_2_ Content

**FIGURE 3:**
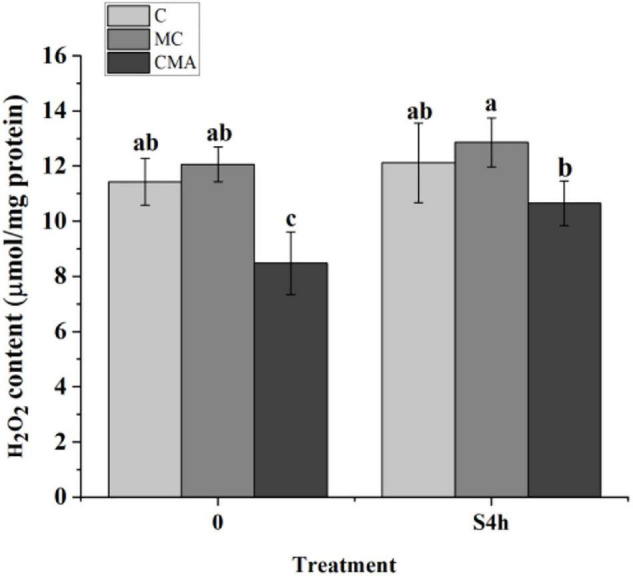
The fluctuation of hydrogen peroxide (H_2_O_2_) content in different treatments of *N. yezoensis*. C, control; MC, Clo + Mev; CMA, Clo + Mev + ABA. The ordinate represented the content of H_2_O_2_. Data were Mean ± SD (*n* = 3). Different letters indicated significant differences (*P* < 0.05).

As showed in Figure 3, the H_2_O_2_ content in the CMA treatment was lower than that in C before hypersaline stress. At S4h, it was up-regulated in the different groups, but there were no significant differences (*P* > 0.05) among C, MC, and CMA. During the hydration process, the H_2_O_2_ level in the MC samples were higher than that in C, and it was lower than that of the control after the CMA treatment.

### Activity Assay of Enzymes Related to the Antioxidant System

**FIGURE 4:**
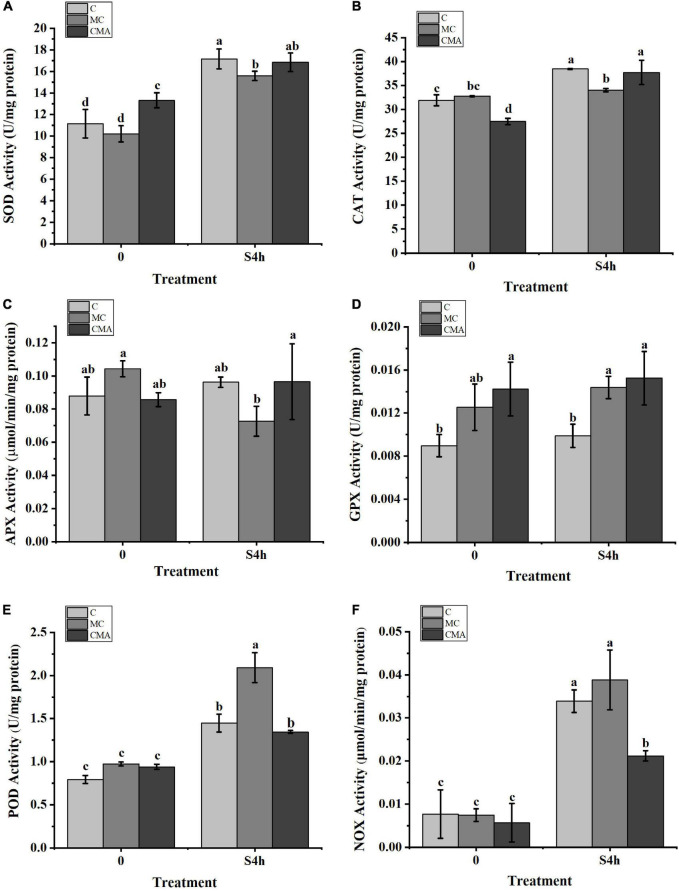
Variation of enzyme activities related to the anti-oxidation system with different inhibitors treatment under hypersaline stress in *N. yezoensis*. **(A)** Superoxide dismutase (SOD); **(B)** catalase (CAT); **(C)** ascorbate peroxidase (APX); **(D)** glutathione peroxidase (GPX); **(E)** peroxidases (POD); **(F)** NADPH oxidase (NOX). The abscissa represented treatment time under hypersaline (120‰) stress conditions. The ordinate represented variation of enzymes activity. C, control; MC, Clo + Mev; CMA, Clo + Mev + ABA. Data were Mean ± SD (*n* = 3). Different letters indicated significant differences (*P* < 0.05).

As showed in Figure 4, SOD, CAT, POD, and NOX activities were increased significantly after high salinity stress. However, high salinity had little effect on APX and GPX activities in the C and CMA samples. With the exception of the down-regulation of APX, other enzyme activities were up-regulated when the MC inhibitors were present under high salinity treatment. More importantly, the addition of exogenous ABA to the MC treatments usually enhanced the activities of SOD, CAT, APX, and GPX under the high salinity conditions.

### Transcriptome Analysis of the Samples Treated With the Different Chemical Treatments

**FIGURE 5:**
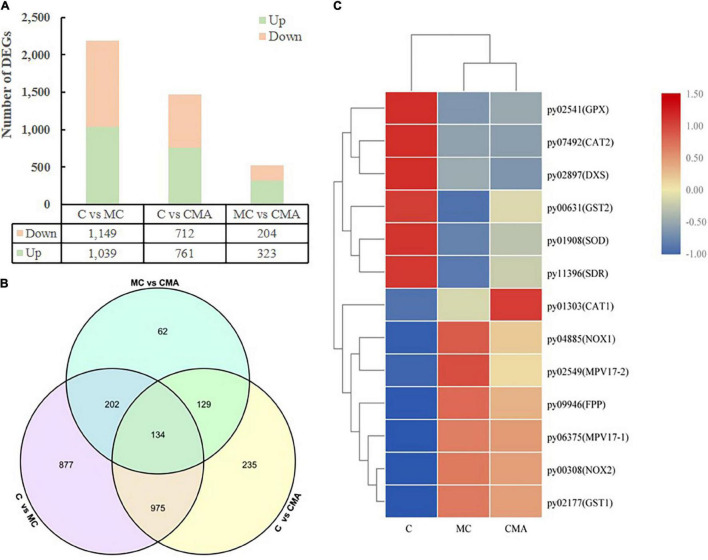
Overview of DEGs among different groups of *N. yezoensis*. **(A)** Column diagram. The horizontal coordinate represented the comparison of different treatment groups, named C vs. MC, C vs. CMA, MC vs. CMA. The vertical coordinate was the number of DEGs. **(B)** Venn diagram. **(C)** Heatmap clustering analysis of DEGs associated with major genes about ABA biosynthesis and antioxidation system.

To investigate the changes in the genes associated with ABA synthesis and the expression of antioxidant enzymes in *N. yezoensis* thalli under 120‰ hypersaline stress, we constructed three cDNA libraries, which were control, MC, and CMA. A total of 11,973 genes were obtained by comparing them to the reference genome for the *N. yezoensis* thallus (Wang et al., 2020). Overall, 2614 DEGs were detected with an FDR < 0.05, log2FC > 1, or log2FC < −1. Compared to C, 1039 and 761 genes were up-regulated, and 1,149 and 712 genes were down regulated in MC and CMA, respectively (Figure 5A). In addition, 323 genes were up-regulated and 204 genes were down-regulated in CMA compared to MC (Figure 5B).

### Differentially Expressed Genes Related to Abscisic Acid Biosynthesis and the Anti-oxidative Enzymes

A KEGG enrichment search identified the pathways related to ABA biosynthesis and the anti-oxidative system. These included the terpenoid backbone biosynthesis (ko00900), glutathione metabolism (ko00480), and peroxisome (ko04146) pathways (Supplementary Table 2). We screened the DEGs related to anti-oxidative enzyme genes and found that the glutathione peroxidase (*GPX*), superoxide dismutase (*SOD*), and catalase (*CAT*) genes had a similar variation tendency after the chemical treatments. Compared to C, the gene expression levels were down-regulated under the MC treatment and up-regulated when exogenous ABA was added (CMA).

The DEGs related to ABA biosynthesis, which belonged to the terpenoid backbone biosynthesis (ko00900) pathway, including 1-dehydro-D-xylulose-5-phosphate synthase (*DXS*), farnesyl pyrophosphate synthase (*FPS*), and xanthoxin dehydrogenase (*XanDH*), were screened. Under the MC inhibitor treatment, the expression levels of the *DXS* and *XanDH* genes were down-regulated, but the addition of exogenous ABA increased their gene expression levels.

A heatmap clustering analysis of the DEGs associated with the anti-oxidative enzymes and ABA biosynthesis is shown in Figure 5C. A total of 15 DEGs were divided into two expression modes in the different treatment groups. The expression levels of the genes were either down-regulated or up-regulated in MC and CMA compared to C (Figure 5C).

### Validation of the Gene Expression Profiles by Quantitative Assays Using Real-Time PCR

**FIGURE 6:**
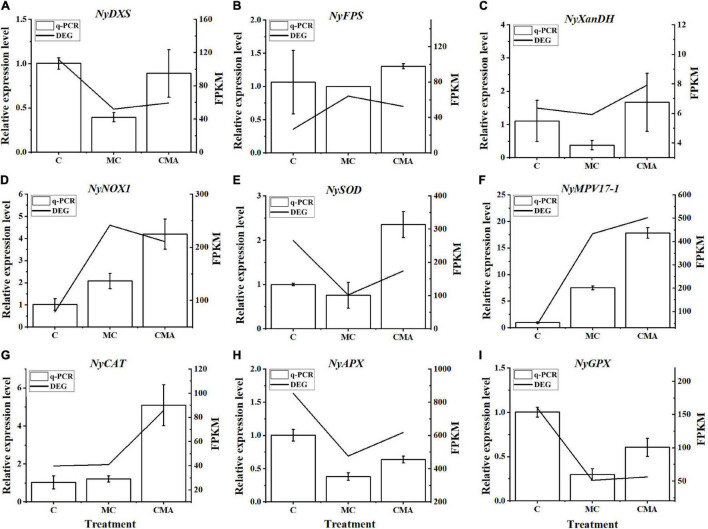
Comparison of qRT-PCR results with those of RNA-Seq data from selected DEGs, including **(A)** *NyDXS* (py02897), **(B)** *NyFPS* (py09946), **(C)** *NyXanDH* (py11396), **(D)** *NyNOX1* (py00308), **(E)** *NySOD* (py01908), **(F)** *NyMPV17-1* (py06375), **(G)** *NyCAT* (py01303), **(H)** *NyAPX* (py06229), and **(I)** *NyGPX* (py02541). The horizontal coordinate represents the treatment of different inhibitors, named C, MC, and CMA. The vertical coordinate represents the relative expression levels of different DEGs and FPKM, respectively. FPKM, fragments per kilobase per million.

A total of nine crucial genes were analyzed by qRT-PCR and the results showed that their expression levels were basically consistent with the transcriptome data, which indicated that the RNA-seq results were reliable (Figure 6).

### Annotation of Key Genes in the Abscisic Acid and Isoprenyl Pyrophosphate Synthesis Pathways

A total of 17 genes associated with the synthesis of ABA and IPP, a precursor of ABA, were identified (Supplementary Table 3). A specific synthesis pathway for ABA and its precursor IPP in *N. yezoensis* is suggested (Supplementary Figure 1) based on our transcriptome data and several previous studies (Hemmerlin et al., 2012; Lohr et al., 2012; Mikami et al., 2016). Among the 17 genes, a total of seven genes were annotated in the MEP pathway for IPP, but only one gene was annotated in the MVA pathway. In addition, nine major genes in the indirect ABA biosynthesis pathway were also annotated (Supplementary Table 3). The DEGs among these included *NyDXS*, *NyFPS*, and *Ny XanDH*.

### Analysis of the Core Components of the Abscisic Acid Signaling Pathway in *N. yezoensis*

All the annotated genes related to the core components of the ABA signaling pathway are summarized in Table 1. Although the PYR/PYL/RCAR-type ABA receptor genes were not found, the other core components, such as protein phosphatase 2C (PP2C), SNF1-related protein kinase 1 (SnRK1), and ABA responsive element-binding transcription factors (AREB/ABF) were identified in *N. yezoensis*. However, a similarity search for the *nyPP2C* sequence in the NCBI database did not match the model *PP2C-A* gene in terrestrial plants. Furthermore, only one SNF1-related protein kinase 1 (SnRK1) transcript, which is believed to be the ancestral member of the SnRK protein family, was annotated in *N. yezoensis*. Additionally, there were 69 different transcripts annotated as the bZIP transcription factor detected, which implied the existence of gene expression regulation by ABA in *N. yezoensis*. And a computer search indicated that *CAT, APX, GPX*, and *POD* harbored the ABRE motif on the upstream 2 kb sequences of the transcription start code, which illustrated that expression of these antioxidant enzymes was regulated by the ABA-signaling pathway.

**TABLE 1:** The core genes of the abscisic acid (ABA) signaling pathway identified in different organisms.

Gene name	Seed plants	Bryophytes	Streptophyte algae	*N. yezoensis*	Gene function description
*PYR/PYL/RCAR*	+	+	*PYL* ortholog	–	Soluble ABA receptor
*PP2C*	*PP2C-A*	*PP2C-A*	*PP2C-A*-like genes	Ancient *PP2C*	Protein phosphatase 2C, releasing SnRK2 from PP2C inhibition
*SnRK2*	+	+	+	*SnRK1*	SNF1-related protein kinase 2, phosphorylates downstream substrates such as ABF
*AREB/ABF*	+	+	+	+	Leucine zipper (bZIP) transcription factor, recognize the ABA-responsive elements in promoters of stress responsive genes

## Discussion

### Inhibitor Treatments Under Hypersaline Conditions

Here, we employed two inhibitor treatments to investigate the synthesis of ABA and the potential ABA regulation of antioxidase expression. The samples were first incubated with the inhibitors for a complete dehydration and rehydration cycle. During the process, the originally ABA contained in the cell is probably consumed due to the stress response by *N. yezoensis*. This eliminated the influence of background ABA on the determinations of various parameters. Furthermore, rehydration with inhibitors ensured that the chemicals were fully absorbed and infiltrated the cell. This favored the accurate determination of ABA content and other determinations during the second round of stress treatments.

### Analysis of a Possible Pathway for Abscisic Acid Synthesis in *N. yezoensis*

*Neopyropia yezoensis* is a type of Archaeplastida and it is supposed to possess ancestral features of primary photosynthetic eukaryotes (Mikami et al., 2016). Usually, the alga are distributed along the intertidal zones and thus it must have evolved special

stress response pathways to survive the extremely variable environments. Although many studies have reported that ABA may play an important role in stress responses by this alga, its endogenous biosynthesis remains poorly understood. A gene survey using EST information showed the existence of genes encoding zeaxanthin epimerase and a xanthoxin dehydrogenase homolog in *N. yezoensis*. Mikami et al. (2016) combined these results with liquid chromatography-mass spectrometry results and strongly suggested that *N. yezoensis* can produce ABA *via* pathways similar to the indirect pathway in terrestrial plants. However, as for the metabolic process prior to carotene synthesis, there have been no previous reports addressed in *N. yezoensis*.

It is well known that carotene is derived from isoprenyl pyrophosphate (IPP) in plants. The synthesis of IPP is prerequisite for ABA biosynthesis, regardless of whether the direct or indirect pathways are used. There are two pathways for the biosynthesis of IPP, the mevalonate (MVA) pathway in the cytoplasm and the methylerythritol phosphate (MEP) pathway in the plastids (Bick and Lange, 2003). Either one or both of these two pathways have been found to exist in cells depending on the organism (Lohr et al., 2012). In brief, it appears that the MEP pathway is essential for plastid-bearing organisms since carotenoids are synthesized in plastids *via* IPP (Lohr et al., 2012). The cytosolic MVA pathway in algae with secondary plastids is considered to be a genetic patchwork, although all the extant algae with secondary plastids that have been investigated contain the MVA pathway (Lohr et al., 2012). Our transcription data revealed the presence of all the key enzyme genes associated with the MEP pathway, among which, the *NyDXS* (py02897) gene was down-regulated when treated with inhibitors (Figure 6). However, only one gene, *NyAACT* of the MVA pathway was identified. Meanwhile, the ABA content decreased under specific inhibition of the MVA pathway by Mev (Figure 2). Meanwhile, the Clo, inhibitor of key *DXS* enzymes in the MEP pathway could effectively reduce the ABA content during high salinity treatment (Figure 2). This suggests that both the MVA and the MEP pathways may be operative in *N. yezoensis*. When the MEP pathway was suppressed, the MVA pathway was activated to compensate for the reduction in IPP supply. Similar findings have also been reported for other phototrophic algae. For example, studies have showed that *Galdieria sulphuraria* and *Cyanidium caldarium* have both the MVA and MEP pathways (Schwender et al., 1997; Disch et al., 1998; Grauvogel and Petersen, 2007; Lohr et al., 2012). Therefore, cooperation between the MVA and MEP pathways in *N. yezoensis* is likely to occur to fine tune the IPP supply.

### Effects of Abscisic Acid on Photosynthetic Efficiency Under Hypersaline Conditions

Both the individual IPP synthesis inhibitors and a combination of the inhibitors significantly reduced the *F*_*v*_/*F*_*m*_ of the samples under high salinity stress conditions. Even in the followed rehydration, the recovery rates of photosynthetic parameters were clearly slower than that of the control, but the treatment samples did eventually recover. The results indicated that treatment with the inhibitors did not cause substantial damage to the seaweed photosynthetic elements and thus the experiments designed in this study can be used to investigate the role of ABA in the regulation of antioxidant enzyme expressions.

Earlier studies showed that ABA promoted the repair of photo-induced inactivation of the photosystem II complex in *C. reinhardtii* under low temperature stress, and that it played a protective role in the photosynthetic system (Saradhi et al., 2000). Our results showed that the ABA synthesis inhibitors significantly reduced the ABA content in *N. yezoensis* (Figure 2) and simultaneously decreased *F*_*v*_/*F*_*m*_ (Figure 1A) under hypersaline conditions. In contrast, the application of exogenous ABA (CMA treatment), significantly increased the ABA content (Figure 2) and slightly increased the *F*_*v*_/*F*_*m*_ values, although the change was not significant (*P* > 0.05) (Figure 1A). Other studies suggested that ABA may not directly improve photosynthetic activity, but may protect the photosynthetic system from damage by ROS (Yoshida et al., 2003). Consequently, ABA may play a vital role in alleviating the effect of hypersaline stress on photosynthesis through the antioxidation system in *N. yezoensis*, but the specific regulatory mechanism needs further study.

### Significance of NADPH Oxidase in the Induction of the Abscisic Acid Signaling Pathway

For a long time, it is not clear whether the synthesis of ROS is necessary for the ABA signaling pathway. While the results in *Arabidopsis* using the mutant illustrated that when the NADPH oxidase gene (*NOX*) was disrupted, the ABA signaling pathway became impaired, whereas exogenous addition of H_2_O_2_ rescued the physiological response in the mutant (Kwak et al., 2003). The central positive inductor of ROS to the ABA signal transduction was constructed. The NOX enzyme is a type of plasma membrane protein and NOX activity may be the main ROS source when the plant is subjected to stress (Lamb and Dixon, 1997). This shows that the transplasma membrane NOX (O_2_^–^ synthases) might be crucially important to ABA signaling.

In this study, two *NOX* transcripts were identified and the results of the qRT-PCR analysis showed that their expression was up-regulated when the inhibitors were applied, but the addition of exogenous ABA reduced the magnitude of the up-regulation compared to that of the control. We also found that the NOX activity analysis produced the same profile as the qRT-PCR results (Figures 4, 6). Based on the current results for NOX alone, we suggested that ABA may down-regulate the expression of the *NOX* gene and reduce the synthesis of O_2_^–^ species. However, it is a paradox since that ROS produced by NOX has been proposed to play a role in the induction of the ABA signaling pathway (Kwak et al., 2003). In fact, ROS scavenging enzyme genes, such as *SOD*, *CAT*, *GPX*, *APX*, and *POD*, may be regulated by ABA and thus contribute to the maintenance of proper redox homeostasis in plant cells (Mittler, 2002; Kwak et al., 2003). Figure 4 shows that the activities of several antioxidase enzymes were up-regulated when exogenous ABA was added compared to the MC inhibitor treatment. Therefore, we propose that the ROS scavenging enzymes, in both C and CMA were induced when the samples were recovered prior to the second round of high salinity stress. The up-regulation of the ROS scavenging enzymes maintained the cell redox homeostasis state and this alleviated the effects of stress on NOX and which decreased the induction of ROS. All these results illustrated the potential interrelationship between *NOX* expression and ABA signaling. On the other hand, the significant upregulation of *NOX* expression and enzyme activity in the inhibitor treatments (MC) demonstrated that the algal cell was in a more severe oxidation state, which triggered the formation of ROS.

### Effects of Abscisic Acid on H_2_O_2_ Production

Hydrogen peroxide is considered to be a byproduct of environmental stress metabolism and is thought to induce cellular damage (Neill et al., 2002; Miller et al., 2010). Here, the H_2_O_2_ content was significantly down-regulated after adding of exogenous ABA compared to the MC and C under the high salinity conditions (Figure 3). It is worth noting that H_2_O_2_ accumulation is also alleviated as ABA content increases in higher plants. For example, Jiang and Zhang (2002) reported that the ABA content in maize leaf cells increased continuously with water stress, while the H_2_O_2_ level increased at first and then decreased. Thus, we speculate that it caused an increase in H_2_O_2_ during the first rehydration period of the algae in the control and the samples with added exogenous ABA. The increase in H_2_O_2_ during the first round of high salinity stress and recovery up-regulated the ROS scavenging system, which constrained H_2_O_2_ accumulation when the samples were subjected to the second round of high salinity treatment. In brief, H_2_O_2_ was induced by ABA at first, but was then removed by the activated antioxidation system. This may be a universal process that is present in all organisms.

### Potential Correlation Between Abscisic Acid and the Antioxidant Enzymes

The effects of ABA on the induction of antioxidant responses in *Pyropia orbicularis* and other intertidal seaweed species have been evaluated and the results showed that ABA regulated the activation of antioxidant enzymes activities during desiccation (Guajardo et al., 2016). Additionally, ABA can significantly increase GR activity in *Platycladus orientalis* (Yao et al., 2020). Similar results were obtained in this study. The CAT, SOD, APX, and GPX activities were up-regulated under hypersaline stress (Figure 4). Furthermore, adding chemicals to inhibit the synthesis of ABA under high salinity conditions decreased antioxidant enzyme activities in the MC treatments compared to C. In contrast, the application of exogenous ABA (CMA) recovered the activities of most antioxidant enzymes. These findings suggest that there is a correlation between ABA and the antioxidant system under high salinity stress in *N. yezoensis.*

Additionally, the transcriptomic data showed that there were two KEGG pathways associated with the oxidation stress response, which were the glutathione metabolism and peroxisome pathways (Supplementary Table 2). There were 10 DEGs related to the anti-oxidative system, including *CAT, SOD, GPX, NOX*, and *glutathione S-transferase* (*GST*) (Figure 5C and Supplementary Table 2). The expression levels of *NyCAT* (py01303) and *NySOD* (py01908) had similar enzyme activity tendencies under hypersaline stress (Figure 6). Previous studies have shown that ABA increases the transcription levels of *Mn-SOD* genes during maize embryo maturation (Zhu and Scandalios, 1994). Our results showed that exogenous ABA could significantly increase expression of the *NySOD* gene in *N. yezoensis* under high salinity stress, which was consistent with the change in SOD enzyme activity (Figures 4A, 6E). Mittler and Zilinskas (1992) found that the cytosolic *ApxI* transcript increased dramatically in response to drought, high temperature, and ABA stress in pea. Williamson and Scandalios (1992) discovered that ABA significantly induced the expression of *Cat-1* in the dark. Our results indicated the *NyAPX* and *NyCAT* genes expressions had the same profiles and were similar to *NySOD.*

However, the change in *NyGPX* (py02541) expression level was inconsistent with its variation in enzyme activity. Glutathione peroxidase is an important antioxidant enzyme that catalyzes the conversion of H_2_O_2_ into water using reduced GSH, thioredoxin (Trx), or other electron donors (Ursini et al., 1995; Bela et al., 2015; Passaia and Margis-Pinheiro, 2015). A number of studies have shown that there are different GPX homologs in organisms (Passaia et al., 2013). Some homologs are up-regulated under certain abiotic stresses, while other homologs may be down-regulated (Zhou et al., 2018). Thus, our transcriptome and qRT-PCR results did not cover all the contributions made by the transcripts with a GPX function. The expression of *NyGPX* was up-regulated when exogenous ABA was added. Therefore, it is reasonable to propose that ABA at least partly regulates *NyGPXs* expression under high salinity stress conditions. The up-regulation of the enzyme activity under the normal conditions could be explained by the induction of ABA during the rehydration prior to high salinity stress with the chemicals.

It is well known that salinity stress can induce ROS accumulation and activate the antioxidant system, which plays an important role in protecting algal cells from oxidative damage (Luo and Liu, 2011; Wang et al., 2019). It has also been shown that ABA is considered to be a common constituent in Rhodophyta where it is involved in regulating physiological processes (Yoshida et al., 2003; Yokoya et al., 2010). Our previous study found that ABA content significantly increased under high salinity stress in *N. yezoensis* (Yu et al., 2018) and that there was also a significant change in antioxidant enzyme activities (Yu et al., 2020). However, whether ABA regulates the expression of antioxidant enzymes in this alga is unknown.

### Signaling Pathway for the Abscisic Acid-Mediated Expression of Antioxidant Enzymes in *N. yezoensis*

Acquisition of the ABA signaling pathway is widely regarded as one of the crucial factors for successful land colonization by terrestrial plants (Komatsu et al., 2020). The mechanism underlying the ABA signaling pathway has been determined in the model plant *A. thaliana*. Under osmotic stress conditions, researchers found that ABA can protect cells from multiple environmental stress factors. Functional genomic studies have suggested that the same signaling components are induced in bryophytes under desiccation conditions as those in angiosperms (Xiao et al., 2018). Indeed, the core ABA signaling components seem to be evolutionarily conserved among land plants (Komatsu et al., 2020). Therefore, the ABA signaling pathway must have existed in a common ancestor of land plants around 400–500 million years ago (Takezawa et al., 2011).

Previous studies have suggested that the ABA receptor gene for PYR/PYL/RCAR was laterally transferred from bacteria to the ancestral genome of the *Zygnematophyceae*, which are believed to be the closest algal relatives of land plants (Wickett et al., 2014; Cheng et al., 2019). Thus, the corresponding pathway in algae before *Zygnematophyceae* was defined as a kind of prototype ABA signaling pathway that might have consisted of different molecules but with similar functions to those in higher plants (Komatsu et al., 2020). Major ABA signaling components other than PYR/PYL/RCAR-type receptor genes have been identified in the streptophyte algae *Klebsormidium nitens* and *Chara braunii* (Hori et al., 2014; Nishiyama et al., 2018). As a core protein in ABA signaling, *PP2C* inhibits *SnRK2* kinase activity by dephosphorylation. It is thought to have emerged early in evolutionary life and is widely distributed in archaea, bacteria, plants, and even animals (Komatsu et al., 2020). During evolution from prokaryotes to terrestrial plants, *PP2C*s diversified and formed different subgroups with increased total gene numbers (Fuchs et al., 2013). The *PP2C* family has been classified into several different groups based on their amino acid sequence characteristics (Fuchs et al., 2013; Bhaskara et al., 2019), of which, group A *PP2C* (*PP2C-A*) may play a crucial role in the ABA signaling pathway. In bryophytes, the PP2C-A gene may have been evolutionarily conserved and could have functioned as a negative regulator that was analogous to that in angiosperms. However, there does not seem to be any *PP2C-A* genes in the algal *PP2C* family except for the *PP2C-A*-like genes found in the chlorophyte genome (Komatsu et al., 2020). In this study, a full length *PP2C* gene was obtained from *N. yezoensis*. However, a similarity search in the NCBI database showed that the *nyPP2C* gene was not an analog of *PP2C-A* but belonged to an ancestral *PP2C* gene.

The SnRK2s phosphorylated AREB transcription factors (AREB/ABFs) and these activated AREB/ABFs recognized the conserved ABA-responsive elements (ABREs) in the promoters of downstream target genes. Based on their C-terminal domain structure, the SnRK family in plants can be defined as SnRK1, SnRK2, and SnRK3 (Hrabak et al., 2003). SnRK1, a SnRK2-like kinase, has also been detected in the green alga *C. reinhardtii*, which is located at the base of the green plant phylogenetic tree (Komatsu et al., 2020). In contrast, only one copy of *SnRK1*-related kinase was found in the genome of *Cyanidioschyzon merolae*, a red alga that emerged before green algae (Komatsu et al., 2020). These results imply that SnRK1 is an older SnRK than SnRK2. Thus, it is reasonable to believe that the association between ancestral PP2C and SnRK1 occurred long before the emergence of PP2C-A/SnRK2. Recent published *Porphyra* genome showed that there was no SnRK proteins with similarity to the SnRK2 subfamily, but it encoded SnRK1 and SnRK3. Furthermore, several members of the SnRK3/CIPK clade contain the similar motifs to the C-terminal domain of SnRK2 (Brawleya et al., 2017). Similarly, there was no *SnRK2* gene was found in our transcriptome data, but one *SnRK1* transcript was annotated in *N. yezoensis*.

Many studies have reported that the AREB/ABFs play a central role in osmotic stress inducible adaptive pathways (Nakashima et al., 2014) and that the transcriptions of stress related genes are enhanced by ABA accumulation. During this process, the most important and well-characterized regulator of AREB/ABFs is the basic leucine zipper (bZIP) transcription factor (Fujita et al., 2013). The named ABRE is constituted by the ACGTGG/TC sequence (Guiltinan et al., 1990; Busk and Pages, 1998). It has been shown that the interaction between the ACGT-box and a so-called coupling element (CE) in higher plants is sufficient to confer ABA induction (Shen et al., 1996; Narusaka et al., 2003) and that an ACGT-box can interact with different CESs, which usually leads to a synergistic effect on the absolute expression levels of the target genes (Shen et al., 1996). A pair of ABREs has been found to form such an ABA-responsive complex (Hattori et al., 2002). In addition, promoter analyses have revealed that multiple ABREs will enhance the expression of the related genes under drought or ABA accumulation (Shen et al., 1996; Narusaka et al., 2003). Several bZIP transcription factors were also identified in our transcriptome. In addition, we also identified at least one ABRE motif in the promoter region of some *N. yezoensis* genes related to the antioxidant system, including *CAT, APX, GPX, POD*, and *NOX*.

Although several core component genes were identified, studies on the ABA signaling pathway in Rhodophyta are still fragmented. Together with the ABA stress response factor, the AREB/ABF-SnRK2 signaling and the ABRE sequence in the promoters of downstream genes are believed to be conserved from bacteria to mammals (Takezawa et al., 2011). This implies the possibility of a mechanistic analogy among the ABA signaling pathways in organisms from different kingdoms. Thus, it is reasonable to assume that ABA signal transduction occurs in *N. yezoensis* and the up-regulation of antioxidase genes under high salinity conditions could be attributed to ABRE-mediated activation.

Although none of the genes for the core components in ABA signaling were identified as DEGs under the MC and the CMA treatments according to our transcriptome analysis. The up-regulation of most antioxidation-related enzymes under high salinity conditions with the addition of exogenous ABA in the inhibitors treatment illustrated the induction of these genes.

## Conclusion

Based on our transcriptome results and analyses of the related molecular information that has been published, we suggest that ABA is synthesized *via* the carotenoid pathway in *N. yezoensis* in a similar manner to terrestrial plants. We also suggest that both the MVA and MEP pathways likely exist simultaneously due to the inhibition of ABA synthesis under the corresponding specific inhibitor treatments. Both the activity and the expression of the antioxidases significantly increased after the addition of exogenous ABA. Except for the PYR/PYL/RCAR-type receptor genes, the other core components of the ABA signaling pathway were identified in *N. yezoensis*. However, these molecules showed evolutionary differences to those in terrestrial plants. This showed that the ABA signaling pathway in *N. yezoensis* is an ancestral one. Taking into account the ABRE motif in the promoter region of genes related to the antioxidant system, we propose that the up-regulation of antioxidase genes under high salinity conditions is mediated by the ABA signaling pathway.

## Data Availability Statement

The data presented in the study are deposited in the Oceanographic Data Center, accession number PAPER2021120050-01.

## Author Contributions

JN and GW conceived and designed the research. JY, ZF, WG, and JN conducted the experiments. JN, JY, and WG analyzed the data. JN, JY, and BY wrote the manuscript. GW checked and revised the manuscript. JN and GW served as the author who were responsible for contact and ensuring communication. All authors contributed to the article and approved the submitted version.

## Conflict of Interest

The authors declare that the research was conducted in the absence of any commercial or financial relationships that could be construed as a potential conflict of interest.

## Publisher’s Note

All claims expressed in this article are solely those of the authors and do not necessarily represent those of their affiliated organizations, or those of the publisher, the editors and the reviewers. Any product that may be evaluated in this article, or claim that may be made by its manufacturer, is not guaranteed or endorsed by the publisher.

## Footnotes

1
http://blast.ncbi.nlm.nih.gov/Blast.cgi

2
https://www.ibm.com

## Supplementary Material

Supplementary Figure 1 Hypothetical synthetic route of ABA and precursor IPP in *N. yezoensis*. Enzymes marked in red indicated that had been annotated in our transcriptome data. The red five-pointed star represents the genes were expressed differentially. GA-3P, glyceraldehyde 3-phosphate; DXP, 1-deoxy-D-xylulose 5-phosphate; MEP, 2-C-Methyl-D-erythritol-4-phosphate; CDP-ME, 4-(cytidines5-diphospho)-2-C-methylerythritol; CDP-MEP, 2-phospho-4-(cytidines5-diphospho)-2-C-methylerythritol; ME-cPP, 2-C-methyl-D-erythritol-2, 4-cyclodiphosphate; HMBPP, hydroxymethyl-2-(E)-butenyl 4-diphosphate; IPP, isopentenyl diphosphate; GPP, Geranyl diphosphate; FPP, Farnesyl diphosphate; GGPP, Geranylgeranyl diphosphate; NSY, neoxanthin synthase; NCED, 9-cis -epoxycarotenoid dioxygenases; AAO, abscisic aldehyde oxidase; ACC, acetoacetyl-CoA; HMGS, 3-hydroxy-3-rnethylglutaryl-CoA synthase; HMG-CoA, 3-hydroxy-3-methylglutaryl-CoA; HMGR, 3-hydroxy-3-methylglutaryl-CoA reductase; MVA, mevalonic acid;. MK, mevalonatekinase; MVAP, mevalonate 5-phosphate; PMK, phosphomevalonate kinase; MVAPP, mevalonate 5-diphosphate; MDC, mevalonate-5-diphosphate decarboxylase. Other abbreviations in this figure were described in Supplementary Table 3.

Supplementary Figure 2 Flow chart of samples treatment.

Supplementary Table 1 Information about the nine genes and primers used in this study.

Supplementary Table 2 Annotate the major KEGG metabolic pathways of DEGs between different sample groups in *N. yezoensis.*

Supplementary Table 3 The genes relevant to ABA synthesis.
