# Supplementary material for: Synthesis of Abscisic Acid in Neopyropia yezoensis and Its Regulation of Antioxidase Genes Expressions Under Hypersaline Stress
Source: Front Microbiol. 2022 Jan 10;12:775710. doi: 10.3389/fmicb.2021.775710 (PMC8784606; doi:10.3389/fmicb.2021.775710)
Supplement: Supplementary file 1 [file Presentation_1.PPTX]

## Slide 1
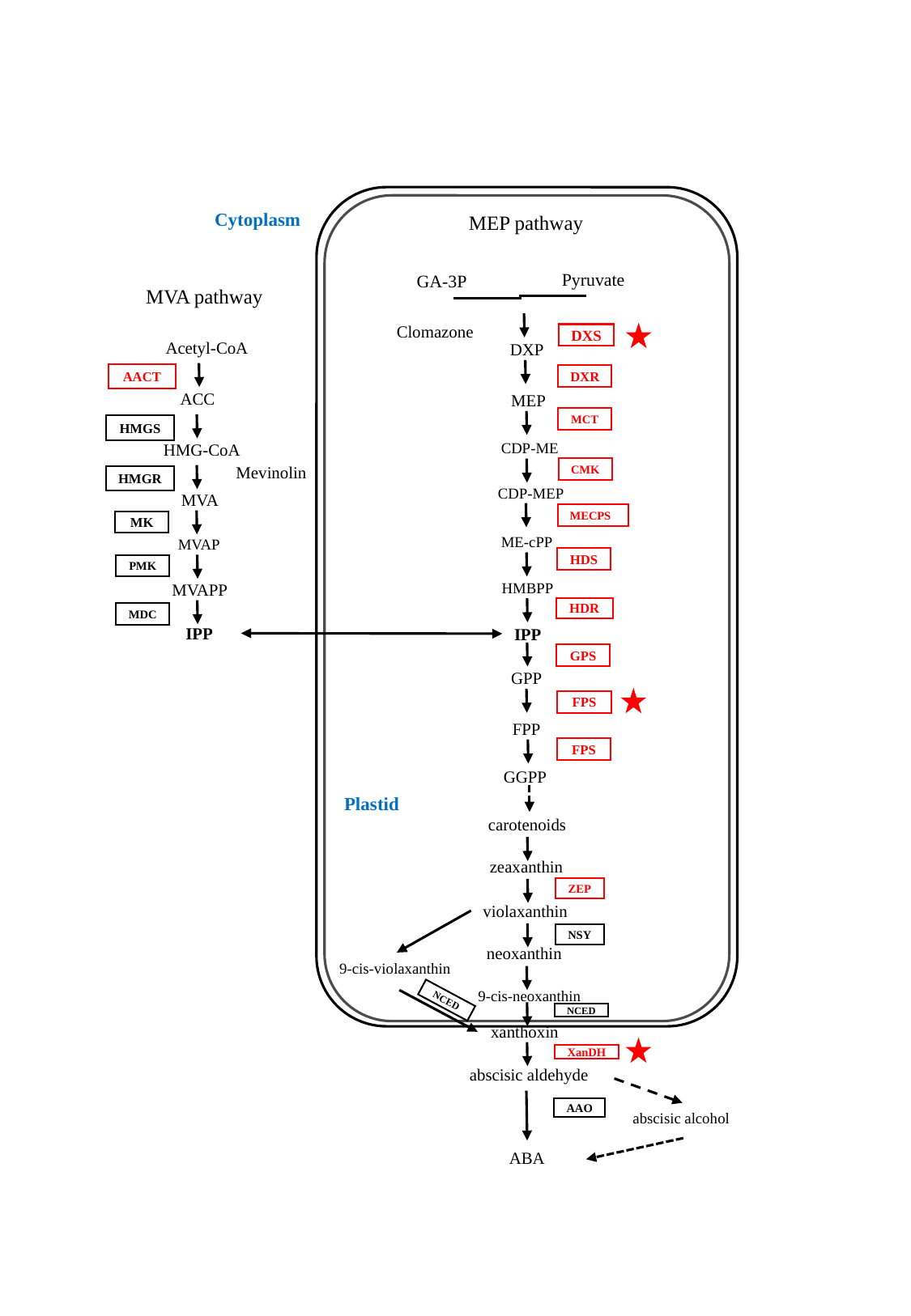

Pyruvate
GA-3P
Clomazone
❌
DXS
MEP
MCT
CDP-ME
CMK
CDP-MEP
MECPS
ME-cPP
HDS
HMBPP
HDR
GPS
GPP
FPS
GGPP
carotenoids
violaxanthin
NSY
9-cis-neoxanthin
XanDH
 abscisic aldehyde
AAO
 ABA
neoxanthin
NCED
 xanthoxin
 abscisic alcohol
9-cis-violaxanthin
NCED
Acetyl-CoA
AACT
ACC
HMGS
HMG-CoA
HMGR
Mevinolin
❌
MVAP
PMK
MVAPP
MDC
IPP
MVA
MK
MEP pathway
MVA pathway
DXP
DXR
IPP
FPS
FPP
zeaxanthin
ZEP
Cytoplasm
Plastid
