# Supplementary figures and images for: Synthesis of Abscisic Acid in Neopyropia yezoensis and Its Regulation of Antioxidase Genes Expressions Under Hypersaline Stress
Source: Front Microbiol. 2022 Jan 10;12:775710. doi: 10.3389/fmicb.2021.775710 (PMC8784606; doi:10.3389/fmicb.2021.775710)

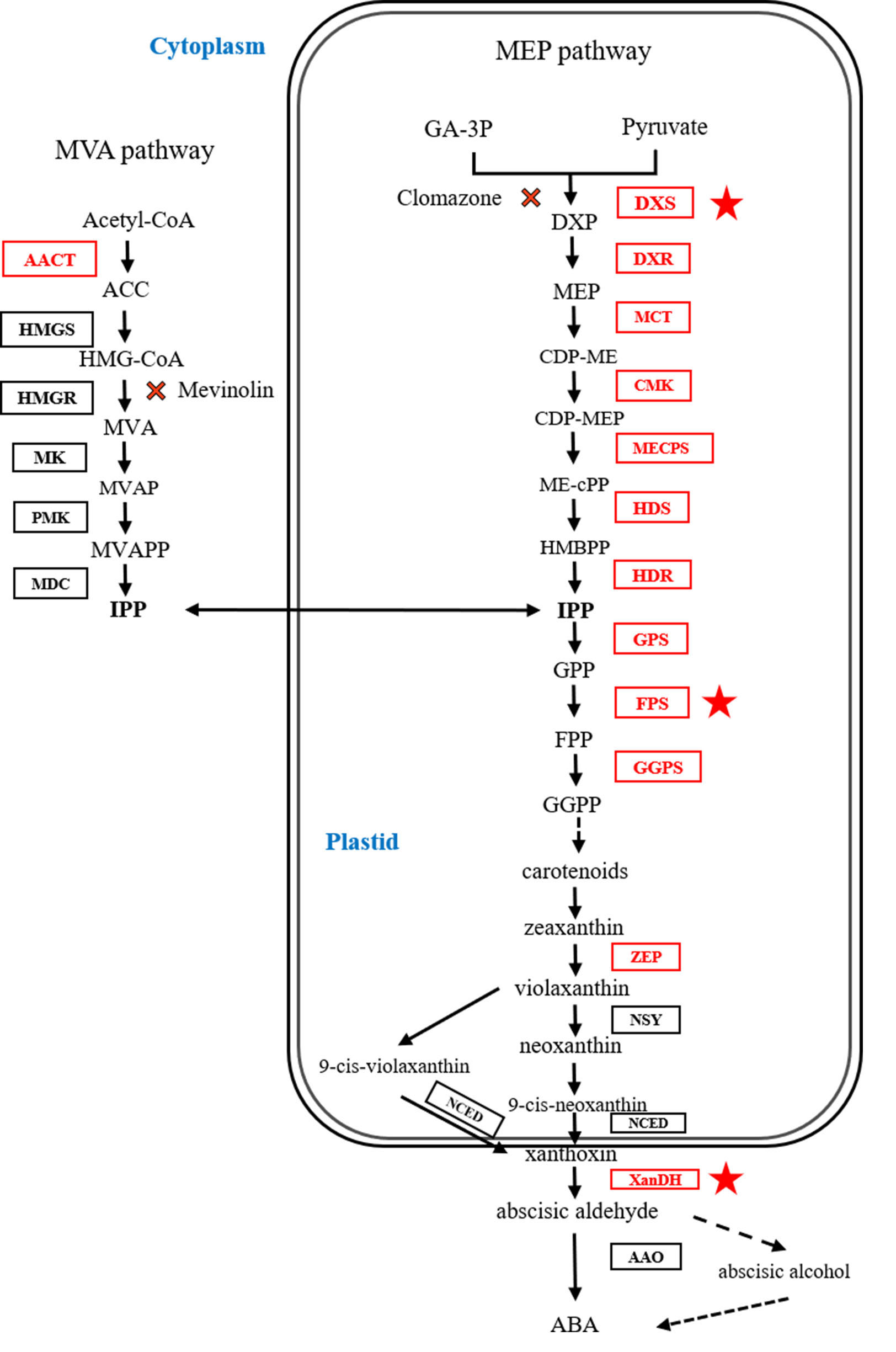

Supplement: Supplementary Figure 1 — Hypothetical synthetic route of ABA and precursor IPP in N. yezoensis. Enzymes marked in red indicated that had been annotated in our transcriptome data. The red five-pointed star represents the genes were expressed differentially. GA-3P, glyceraldehyde 3-phosphate; DXP, 1-deoxy-D-xylulose 5-phosphate; MEP, 2-C-Methyl-D-erythritol-4-phosphate; CDP-ME, 4-(cytidines5-diphospho)-2-C-methylerythritol; CDP-MEP, 2-phospho-4-(cytidines5-diphospho)-2-C-methylerythritol; ME-cPP, 2-C-methyl-D-erythritol-2, 4-cyclodiphosphate; HMBPP, hydroxymethyl-2-(E)-butenyl 4-diphosphate; IPP, isopentenyl diphosphate; GPP, Geranyl diphosphate; FPP, Farnesyl diphosphate; GGPP, Geranylgeranyl diphosphate; NSY, neoxanthin synthase; NCED, 9-cis -epoxycarotenoid dioxygenases; AAO, abscisic aldehyde oxidase; ACC, acetoacetyl-CoA; HMGS, 3-hydroxy-3-rnethylglutaryl-CoA synthase; HMG-CoA, 3-hydroxy-3-methylglutaryl-CoA; HMGR, 3-hydroxy-3-methylglutaryl-CoA reductase; MVA, mevalonic acid;. MK, mevalonatekinase; MVAP, mevalonate 5-phosphate; PMK, phosphomevalonate kinase; MVAPP, mevalonate 5-diphosphate; MDC, mevalonate-5-diphosphate decarboxylase. Other abbreviations in this figure were described in Supplementary Table 3. [file Image_1.JPEG]

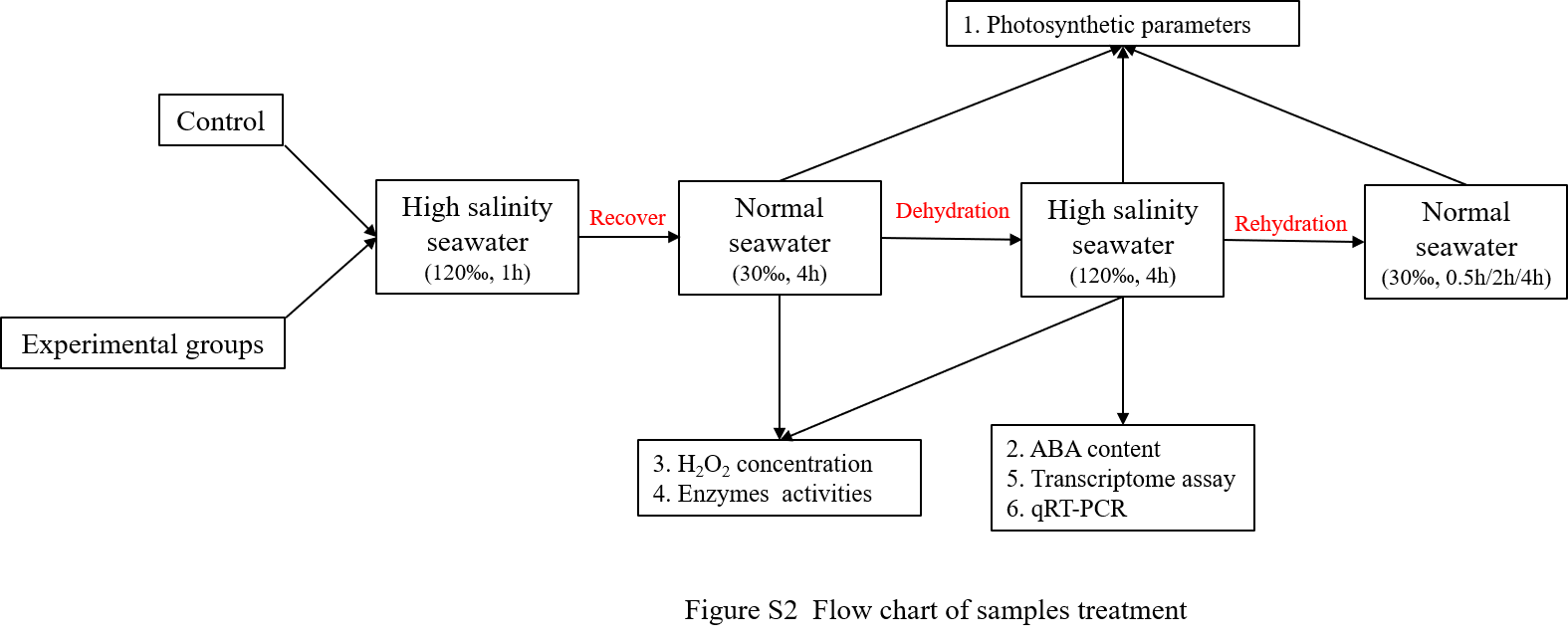

Supplement: Supplementary Figure 2 — Flow chart of samples treatment. [file Image_2.PNG]
