# Supplementary material for: Synthesis of Abscisic Acid in Neopyropia yezoensis and Its Regulation of Antioxidase Genes Expressions Under Hypersaline Stress
Source: Front Microbiol. 2022 Jan 10;12:775710. doi: 10.3389/fmicb.2021.775710 (PMC8784606; doi:10.3389/fmicb.2021.775710)
Supplement: Supplementary Table 1 — Information about the nine genes and primers used in this study. [file Table_1.DOCX]

Supplementary Table 1 Information about the nine genes and primers used in this study

| Gene_name | Gene function description | Primer sequence (5′-3′) | Product size (bp) |
| --- | --- | --- | --- |
| *Actin* | Actin | CAAGCAGAAGGGCATCAT  CCGAGTAGAAAGCGTGGT | 164 |
| *PyDXS* | 1-deoxy-D-xylulose 5-phosphate synthase | GCGGGCAAGAACAATGAG  GGGAAGAGGCGGGAGAAG | 272 |
| *PyFPS* | Farnesyl pyrophosphate synthase | CACTGCTGCGTTTACCCC  CAATGTCCGTGCCGACCT | 236 |
| *PyXanDH* | xanthoxin dehydrogenase | CGTTTGCCGTCAATGTGG  GTGCTCTGAAGGTCCCCC | 202 |
| *PyNOX1* | NADPH oxidase 1 | GAGCACCCCCCTGTCCATC  GGCGAGCACAAATCCCGTC | 205 |
| *PySOD* | Superoxide dismutase | TCAGCAGCACTCCCAACCA  GTCTTCCACCACGACGCAA | 151 |
| *PyMPV17-1* | *MPV17-1* | TGGATTGCCTTTTTGAGC  AGGATAGGAGGGTGGGTC | 181 |
| *PyCAT* | Catalase | GTCTGAGGGCATTTGGGAT  TACGCGTGGACGCTGTTTC | 249 |
| *PyAPX* | Ascorbate peroxidase | CACCTGCGCGACATCTTT  CGGGCGTCTCCTTCATAA | 177 |
| *PyGPX* | Glutathione peroxidase | GGTGCGAATGCACGACACC  CCAGCCCCATCCCAAACAG | 191 |

Supplementary Table 2 Annotate the major KEGG metabolic pathways of DEGs between different sample groups in *N.yezoensis*

| KEGG  Pathway | Pathway ID | Gene name  (Gene ID） | | CS | | MC | | CMA | |
| --- | --- | --- | --- | --- | --- | --- | --- | --- | --- |
|  |  |  |  | Expression levels | | | | | |
| Terpenoid backbone biosynthesis | ko00900 | *PyDXS* (py02897) | | 112.64 | | 55.30 | | 56.97 | |
|  |  | *PyFPS* (py09946) | | 26.74 | | 63.9 | | 52.48 | |
|  |  | *PyXanDH* (py11396) | | 6.355 | | 5.92 | | 7.91 | |
| Glutathione metabolism | ko00480 | *PyGPX*  (py02541) | | 159.64 | | 51.23 | | 56.05 | |
|  |  | *PyNOX1* (py00308) | | 78.14 | | 241.43 | | 210.78 | |
|  |  | *PyNOX2* (py04885) | | 104.24 | | 200.49 | | 160.05 | |
|  |  | *PyGST1*  (py02177) | | 50.53 | | 112.80 | | 102.24 | |
|  |  | *PyGST2*  (py00631) | | 21.69 | | 10.673 | | 14.33 | |
| Peroxisome | ko04146 | *CAT1*  (py01303) | | 39.73 | | 40.97 | | 85.4 | |
|  |  | *CAT2*  (py01303) | | 1.05 | | 0.033 | | 0.023 | |
|  |  | *PySOD*  (py01908) | | 307.96 | | 146.11 | | 177.34 | |
|  |  | *PyMPV17-1*  (py06375) | 46.00 | | 432.77 | | 685.26 | |  |
|  |  | *PyMPV17-2*  (py06375) | 1.09 | | 4.199 | | 2.50 | |  |

Supplementary Table 3 The genes relevant to ABA synthesis

| ABA synthesis pathway | Gene_name | Gene_ID | | Gene function description |  |
| --- | --- | --- | --- | --- | --- |
| MEP pathway | *PyDXS* | py09513 | | 1-deoxy-d-xylulose 5-phosphate synthase | |
|  | *PyDXR* | py08594 | | 1-deoxy-D-xylulose-5-phosphate reductoisomerase | |
|  |  | py08595 | |  |  |
|  | *PyMCT* | py02211 | | 2-C-methyl-d-erythritol 4-phosphate cytidylyltransferase | |
|  | *PyCMK* | py04145 | | 4-(cytidine 5-diphospho)-2-C-methyl-d-erythritol kinase | |
|  | *PyMECPS* | py00887 | | 2-C-methyl-d-erythritol 2,4-cyclodiphosphate synthase | |
|  | *PyHDS* | py03858 | | 4-hydroxy-3-methylbut-2-en-1-yl diphosphate synthase | |
|  | *PyHDR* | py08540 | | 4-hydroxy-3-methylbut-2-en-1-yl diphosphate reductase | |
| MVA pathway | *PyAACT* | py07283 | acetoacetyl-CoA thiolase | |  |
| carotenoid pathway | *PyGPS* | py00011 | geranyl diphosphate synthase | |  |
|  | *PyFPS* | py09946 | Farnesyl pyrophosphate synthase | |  |
|  | *PyGGPS* | py08418 | geranylgeranyl pyrophosphate synthase 7 | |  |
|  | *PyPSY* | py02040 | phytoene synthase | |  |
|  | *PyPDS* | py05365 | phytoene desaturase | |  |
|  | *PyZDS* | py03957 | carotene desaturase | |  |
|  | *Py* *LCYb* | py04414 | lycopene β-cyclase | |  |
|  | *PyZEP* | py03929 | zeaxanthin epoxidase | |  |
|  | *PyXanDH* | py11396 | xanthoxin dehydrogenase | |  |
